# Absent Exercise-Induced Improvements in Fat Oxidation in Women With Polycystic Ovary Syndrome After High-Intensity Interval Training

**DOI:** 10.3389/fphys.2021.649794

**Published:** 2021-03-24

**Authors:** Sofie Lionett, Ida Almenning Kiel, Ragnhild Røsbjørgen, Stian Lydersen, Steen Larsen, Trine Moholdt

**Affiliations:** ^1^Department of Circulation and Medical Imaging, Norwegian University of Science and Technology, Trondheim, Norway; ^2^Department of Obstetrics and Gynecology, St. Olav's Hospital, Trondheim, Norway; ^3^Department of Mental Health, Regional Centre for Child and Youth Mental Health and Child Welfare, Norwegian University of Science and Technology, Trondheim, Norway; ^4^Department of Biomedical Sciences, University of Copenhagen, Copenhagen, Denmark; ^5^Clinical Research Centre, Medical University of Bialystok, Bialystok, Poland

**Keywords:** endocrinology, metabolic flexibility, exercise, cardiorespiratory fitness, insulin resistance, mitochondrial respiration, adipose tissue, cell size

## Abstract

**Background:** Polycystic ovary syndrome (PCOS) and metabolic inflexibility are linked to insulin resistance, and women with PCOS appear to be metabolic inflexible in the rested, insulin-stimulated state. Exercise training is a primary lifestyle intervention in PCOS. Exercise training improves whole-body fat oxidation during submaximal exercise in healthy women, yet little is known about the effect on this outcome in women with PCOS.

**Methods:** We measured whole-body fat oxidation rates during sub maximal exercise before and after 16 weeks of high-intensity interval training (HIT) in women with PCOS randomly allocated to either: low- or high-volume HIT (*n* = 41; low-volume HIT, 10 × 1 min work bouts at maximal, sustainable intensity and high-volume HIT, 4 × 4 min work bouts at 90–95% of maximal heart rate) or non-exercise control (*n* = 23), and in women without PCOS (Non-PCOS) allocated to low- or high volume HIT (*n* = 15). HIT was undertaken three times weekly. In a subset of women with and without PCOS, we measured mitochondrial respiration in abdominal and gluteal subcutaneous adipose tissue using high-resolution respirometry, as well as fat cell sizes in these tissues.

**Results:** At baseline, women with PCOS had lower whole-body fat oxidation and mitochondrial respiration rates in abdominal adipose tissue compared to Non-PCOS. Peak oxygen uptake (mL/min/kg) increased in women with PCOS (~4%, *p* = 0.006) and Non-PCOS (~6%, *p* = 0.003) after 16 weeks of HIT. Whole-body fat oxidation only improved in Non-PCOS after HIT. No changes were observed in mitochondrial respiration and cell size in abdominal and gluteal adipose tissue after HIT in either group of women.

**Conclusion:** We observed exercise-induced improvements in whole-body fat oxidation during submaximal exercise in Non-PCOS, but not in women with PCOS, after 16 weeks of HIT, suggesting metabolic inflexibility in women with PCOS.

**Clinical Trial Registration:** www.clinicaltrials.gov, identifier NCT02419482 and NCT02943291.

## Introduction

Polycystic ovary syndrome (PCOS) is the most common endocrine disorder in reproductive-age women, affecting up to 13% of women globally (Bozdag et al., 2016). PCOS is associated with increased risk of infertility, insulin resistance, type 2 diabetes and cardiovascular diseases (Moran et al., 2010; Teede et al., 2010; De Groot et al., 2011). Despite the high prevalence and adverse health complications of PCOS, the underlying mechanisms and optimal treatment are still unclear. Insulin resistance and the compensatory hyperinsulinemia is proposed to play a central role in the pathophysiology, contributing to the metabolic and reproductive features of PCOS (Diamanti-Kandarakis and Papavassiliou, 2006).

Metabolic flexibility is the ability to alter substrate use in response to a physiological stimulus, including the transition from fasting to fed states/insulin stimulation or exercise (Goodpaster and Sparks, 2017). Metabolic inflexibility, characterized by distorted nutrient sensing, blunted substrate switching, and impaired energy homeostasis, is linked to insulin resistance (Goodpaster and Sparks, 2017). Women with PCOS appear to have higher metabolic inflexibility in the rested, insulin-stimulated state compared to unaffected women (Rimmer et al., 2020). Previous studies have suggested that the insulin resistance observed in PCOS may be linked to aberrant adipose tissue morphology and function including enlarged adipocytes and decreased insulin-stimulated rates of glucose utilization in adipocytes (Dunaif et al., 1992; Manneras-Holm et al., 2011).

Lifestyle modification including regular physical activity is regarded as first-line therapy in women with PCOS (Teede et al., 2018). Exercise training induces a multitude of positive, health-related outcomes in women with PCOS (Kite et al., 2019), and superior effects of vigorous intensity exercise compared to moderate intensity training, have been observed among women with PCOS (Greenwood et al., 2016; Patten et al., 2020).

The aims of this study were first to compare whole-body fat oxidation during submaximal exercise, cell size and mitochondrial respiration of adipose tissue in women with and without PCOS, and second to assess the response to 16 weeks of HIT.

## Materials and Methods

### Study Design

The present study is a secondary analysis of a two-center, randomized controlled trial; IMProving Reproductive function in women with Polycystic OVary syndrome with high-intensity Interval Training [IMPROV-IT (Kiel et al., 2020); ClinicalTrials.gov identifier: NCT02419482] conducted at the Norwegian University of Science and Technology (NTNU) in Trondheim, Norway and the Australian Catholic University (ACU) in Melbourne, VIC, Australia, and a randomized, uncontrolled trial undertaken at NTNU [The Adipose Tissue Function and Response to Exercise Training in Women With and Without Polycystic Ovary Syndrome trial (HIT-FAT); ClinicalTrials.gov identifier: NCT02943291]. The detailed study design for the IMPROV-IT trial has been published elsewhere (Kiel et al., 2020). In brief, after stratification for BMI < or ≥ 27 kg/m^2^ and study center, women with PCOS were randomized in a 1:1:1 manner to 16 weeks of semi-supervised HIT or a no-exercise control group: (1) Low-volume HIT (LV-HIT), (2) High-volume HIT (HV-HIT), or (3) Non-exercise (Non-Ex).

In the HIT-FAT trial, women without PCOS were selected as a control group, and individually matched by age (± 5 years) and BMI (± 2 kg/m^2^) to women with PCOS in the IMPROV-IT trial. Women without PCOS were randomly allocated (1:1), after stratification for BMI < or ≥ 27 kg/m^2^, to 16 weeks of semi-supervised LV-HIT or HV- HIT. For the purpose of the analyses in this report and because no differences were observed between the LV-HIT and HV-HIT groups, we pooled the LV-HIT and HV-HIT groups into one group for women with PCOS (PCOS HIT) and one group for women without PCOS (Non-PCOS HIT).

### Ethical Approval

The studies were performed according to the Helsinki declaration and approved by The Regional Committee for Medical and Health Research Ethics in Central Norway (REK-midt 2015/468 and 2016/545), and the ACU Human Research Ethics Committee (2017-260H). Participants were informed about the experiments and potential risks verbally and in writing before their written consent was obtained.

### Participants

Sixty-four previously inactive women with PCOS and 15 previously inactive women without PCOS were included in this study. PCOS was defined according to the Rotterdam criteria (Rotterdam, 2004), with at least two of the following three features present: polycystic ovary morphology (12 or more 2–9 mm follicles or >10 ml in volume in at least one ovary), hyperandrogenism (either clinical signs such as acne or hirsutism, or biomedical), and/or oligo/amenorrhea. Hirsutism was defined as a modified Ferriman-Gallwey score of ≥8 (Ferriman and Gallwey, 1961). The PCOS diagnosis was ruled out in all the women without PCOS as they were normally menstruating, with no evidence of hyperandrogenism or polycystic ovaries.

To be eligible for inclusion, the women had to be between 18 and 45 years old and were excluded if they were undertaking regular endurance training ≥ 2 sessions/week, had any cardiovascular diseases or endocrine disorders, were pregnant, had been breastfeeding within the last 24 weeks, or if they were using hormonal contraceptives, insulin sensitizers or drugs known to affect gonadotropin or ovulation (with a washout period of 3 months prior to inclusion).

### Interventions

The exercise training was semi-supervised during the 16 weeks intervention period, and participants attended at least one weekly supervised training session, with the opportunity to perform the two remaining weekly sessions either supervised at the study centers or unsupervised (total of three exercise sessions per week).

The exercise training protocols have been described previously (Kiel et al., 2020). Briefly, participants walked or ran on treadmills during the supervised exercise sessions, while they could choose to perform the unsupervised exercise sessions walking or running on treadmills or outdoors. The LV-HIT protocol consisted of 10 × 1 min work bouts at the maximal intensity the participants could sustain, interspersed by 1 min of passive recovery or low-intensity walking. The HV-HIT protocol comprised 4 × 4 min work bouts at an intensity corresponding to 90–95% maximal heart rate (HR_max_) interspersed by 3 min active recovery at ~70% of HR_max_. All training sessions included 10 min warm-up and 3 min cool-down. Participants wore HR monitors (Polar M400) during all exercise sessions, and registered their exercise sessions via an online exercise-training diary (Polar Flow) which the researchers had access to. Thereby, the researchers could supervise adherence to the protocols.

Women with PCOS assigned to the Non-Ex group were advised to continue their habitual physical activity and informed about the current recommendations of at least 150 min weekly of moderate intensity physical activity. All participants were instructed to maintain their habitual diet throughout the intervention period, and dietary intake was controlled using a 4-day diet recall at baseline and in the last week of the intervention. Physical activity level was monitored using activity monitors (Sensewear Armband, APC Cardiovascular, UK) for 5 days at baseline and during the last week of the intervention period.

### Outcomes

**Figure 1:**
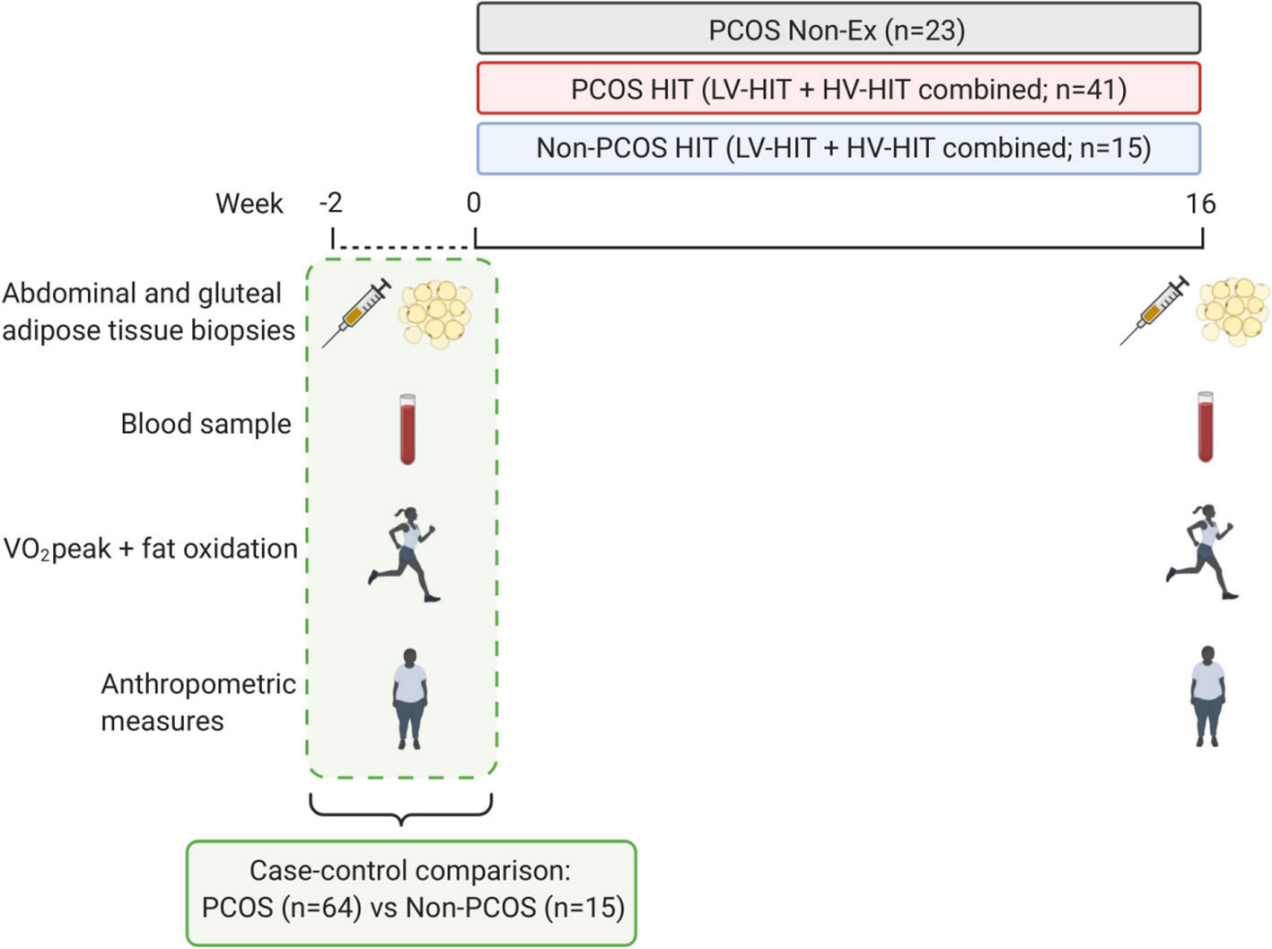
Study protocol. The testing days included: abdominal and gluteal subcutaneous adipose tissue biopsies, fasting blood sampling, VO_2_peak, submaximal exercise test to measure fat oxidation rates, body composition measures, and waist and hip circumference. The adipose tissue was used to measure mitochondrial respiration and cell size. The testing days were repeated after a 16 weeks intervention period of either performing high-intensity interval training or no exercise. PCOS, Polycystic ovary syndrome; Non-Ex, no exercise; HIT, high-intensity interval training; LV-HIT, low-volume high-intensity interval training; HV-HIT, high-volume high-intensity interval training; VO_2_peak, peak oxygen uptake. Created with BioRender.com.

Figure 1 displays an overview of the study protocol. Outcomes were assessed at baseline and after 16 weeks of intervention. All assessments were performed in the early follicular phase (day 1–7 after first bleeding) of the participants' menstrual cycle in women with a regular menstrual cycle while women with oligo/amenorrhea were tested independent of their cycle day.

Participants visited the laboratory on three separate occasions at baseline and after 16 weeks. On the first visit, participants performed an incremental test to exhaustion on a treadmill. We used an individualized protocol to measure peak oxygen uptake (VO_2_peak) and maximal heart rate (HR_max_); after 10 min warm-up and 3 min at moderate intensity, the treadmill speed or inclination was increased every 1–2 min by 0.5–1.0 km/h or 1–2% until volitional exhaustion. The recorded HR_max_ was used to calculate the intensity for the HIT sessions (Berglund et al., 2019).

On the second visit, participants returned to the laboratory after an overnight fast (≥12 h) and refraining from exercise >48 h prior. In Norway, body composition was estimated using bioelectrical impedance analysis (InBody 720, Biospace CO, Korea) while Dual-energy X-ray absorptiometry (DXA; GE Lunar iDXA Pro, Encore software version 16, General Electric, Boston, MA, USA) was used in Australia. Body composition was estimated using the same method in each individual at each timepoint. Waist and hip circumference were measured in duplicate to the nearest 0.5 cm. A resting blood sample was collected in a 5 mL serum tube and rested for 30 min in room temperature before it was spun at 2,200 rpm at 20°C for 10 min. Serum was collected and stored at −80°C for further analysis. On the same day, abdominal and gluteal subcutaneous adipose tissue biopsies were obtained using a 14-gauge needle under local anesthesia (1% Xylocaine) excising ~300–500 mg of tissue from each depot. The adipose tissue was washed on gauze with saline, and capillaries and connective tissue were removed. Approximately 80 mg was allocated for immediate analysis of mitochondrial respiration using high-resolution respirometry (Oxygraph-2K, Oroboros, Innsbruck, Austria). Some of the remaining tissue (~200–300 mg) was snap-frozen in liquid nitrogen and stored at −80°C for later analysis, whereas the rest was immediately fixed in phosphate-buffered formalin for subsequent fat cell size analysis. We were not always able to obtain adipose tissue biopsies from both depots and/or unable to excise enough adipose tissue for all analyses, and this is why the number of samples is lower in mitochondrial respiration and morphology measurements.

On the third visit and following an overnight fast, participants performed a submaximal test on a treadmill. The protocol included 20 min warm-up without gas sampling, followed by 20 min of steady-state workload at 60% of VO_2_peak with sampling of the expired gas. Participants recorded their dietary intake the day prior to baseline testing and repeated this diet before the subsequent measures at 16 weeks. Fat oxidation rates (g/min) was calculated from 5 min of steady oxygen uptake during the last 10 min of the test; 1.695 × VO_2_-1.701 × VCO_2_, where VO_2_ is oxygen uptake and VCO_2_ is expired carbon dioxide (Jeukendrup and Wallis, 2005).

#### Blood Analyses

Plasma glucose concentrations were determined using a Roche Moduclar P (Roche, Switzerland) and serum insulin concentrations were measured in duplicate using an enzyme-linked immunosorbent assay (ELISA; IBL-International, Germany). We estimated insulin resistance using the Homeostatic Model Assessment for Insulin Resistance (HOMA-IR); fasting serum insulin (μIU/mL) × fasting plasma glucose (mmol/L) divided by 22.5 (Matthews et al., 1985). HOMA-IR < 2.5 was considered normal while HOMA-IR ≥ 2.5 indicated insulin resistance. These cut-off points for insulin resistance have been used previously in the PCOS literature (Chuang et al., 2015). HbA1c was analyzed on Tosoh Automated Glycohemoglobin Analyzer HLC-723G8 version 5.24 in Norway and using Cobas b 101 system (Roche Diagnostics) in Australia. We measured testosterone and sex-hormone-binding globulin (SHBG) concentrations in women with PCOS. Total testosterone concentrations were analyzed with Agilent 1290 with 6410 Triple Quad LC/MS-MS detector (Agilent, Santa Clara, United States) SHBG concentrations were analyzed with Advia Centaur XPT (Siemens, Erlangen, Germany).

#### Mitochondrial Respiration

Mitochondrial respiration in abdominal and gluteal subcutaneous adipose tissue was measured in a subset of participants. We measured mitochondrial respiration in the abdominal adipose tissue from 10 non-PCOS women and 16 women with PCOS, and in gluteal adipose tissue from 14 non-PCOS women and 18 women with PCOS.

Mitochondrial respiration measurements were carried out in duplicate in ~40 mg abdominal and gluteal subcutaneous adipose tissue using high-resolution respirometry (Oxygraph-2K respirometer; Oroboros, Innsbruck, Austria). Each of the two chambers of this instrument contained 2 mL Buffer Z (1 mM EGTA; 5 mM MgCl_2_6H_2_O; 105 mM K-Mes; 10 mM KH_2_PO_4_; 5 mg/mL BSA; pH 7.1 at 37°C). All measurements were performed at 37°C and oxygen concentrations > 100 nmol/mL.

We used a protocol modified from Kraunsoe et al. (2010). Amplex Ultra Red (10 μM), Peroxidase from horseradish (HRP; 1 U/mL) and Superoxide dismutase (SOD; 5 U/mL) were titrated into the chambers and stabilized before ~40 mg adipose tissue was added. Digitonin (2 μM) was added to the chambers to permeabilize the cells and baseline respiration was measured before substrates and inhibitors were added. Malate (2 mM) and Octanoyl carnitine (1.5 mM) were added together for measurement of a stable respiration with electron input through complex I and from ß-oxidation. Thereafter, Adenosine diphosphate (ADP; 5 mM) was added to stimulate phosphorylation and obtain maximum electron flow through electron transporting flavoprotein. Pyruvate (5 mM) and Glutamate (10 mM) were then added to measure state 3 respiration specific to complex I, followed by addition of Succinate (10 mM) for measurements of the maximal coupled state 3 respiration. Oligomycin (2.5 mM) was added as an Adenosine 5'-triphosphate (ATP)-synthase inhibitor, and thereafter Carbonyl cyanide m-chlorophenylhydrazone (CCCP) was titrated (starting with 2 μM and followed by steps of 1 μM) to obtain maximal uncoupled respiration. Chambers were then opened slightly for 2–3 min to increase the oxygen concentration in the chamber and avoid oxygen concentrations < 100 nmol/mL for the rest of the protocol. Rotenone (0.5 μM) was added to inhibit the flow of electrons through complex I, followed by Malonic acid (MnA; 5 mM) to inhibit complex II, and later Antimycin A (2.5 μM) to inhibit complex III. Finally, Ascorbate (2 mM) and Tetramethyl-p-phenylenediamine dihydrochloride (TMPD; 0.5 mM) were added together to donate electrons directly to cytochrome c oxidase (COX), and shortly after Sodium azide (≥100 mM) was added as a COX inhibitor. Hydrogen peroxide (H_2_O_2_; 0.1 μM) was added before the following steps: adipose tissue, digitonin, ADP, Oligomycin, Rotenone and Ascorbate to measure H_2_O_2_ flux. All substrate and inhibitor concentrations are final concentrations.

#### Adipose Tissue Cell Size

Adipose tissue was fixated in phosphate-buffered formalin immediately after sampling, before it was embedded in paraffin and cut into 4 μm sections. These sections were mounted on glass slides and dried at 37°C overnight in an incubator. The sections were stained with CD68 [Mouse monoclonal anti-CD68 (Dako, M0814)] in a Dako Autostainer [EnVision™ + Systems HRP (DAB) Mouse (Dako, K4007)]. Digital images were captured with an EVOS FL Auto 2 Imaging System (ThermoFisher Scientific, USA) at x10 objective. Images from the EVOS were analyzed with Fiji (Schindelin et al., 2012). The investigators were blinded for group (PCOS/Non-PCOS, HIT/Non-Ex) and time-point (baseline/16 weeks). The area of ≥200 adipocytes was measured per sample. Fat cell sizes in abdominal adipose tissue was measured from 7 Non-PCOS women and 22 women with PCOS, and in gluteal adipose tissue from 13 Non-PCOS women and 29 women with PCOS.

### Statistical Analysis

We calculated the sample size in the IMPROV-IT trial based on the primary outcome; menstrual frequency (Kiel et al., 2020). A one-way analysis of variance test with three groups with a 5% level of significance, a standard deviation of 2 menstrual cycles/year and statistical power of 0.80 gave a target study population of 48 women to detect an increase of three menstrual cycles during a 12-month period. We added 15% to the sample size owing to the non-normality of menstrual frequency, and another 15% to allow for expected dropout, and aimed to include 64 women with PCOS. We did not perform an *a priori* sample size calculation for the outcomes reported in the present paper, but the number of participants in the groups included in our study corresponds to previous studies with similar outcomes (Despres et al., 1984; Talanian et al., 2007; Perry et al., 2008; Larsen et al., 2015; Dohlmann et al., 2018). The number of Non-PCOS women from whom we measured abdominal fat cell size (*n* = 7) was lower compared with previous studies (Despres et al., 1984; Manneras-Holm et al., 2011).

To determine between-group differences in the LV-HIT and HV-HIT groups (for PCOS and Non-PCOS women, respectively) in all reported outcomes, we used linear mixed models with participants as random factor and the effect of time and group allocation as fixed effects with these levels: PCOS baseline, Non-PCOS baseline, PCOS Non-Ex post intervention, PCOS LV-HIT post intervention, PCOS HV-HIT post intervention, Non-PCOS LV-HIT post intervention, and Non-PCOS HV-HIT post intervention. Since there were no between-group differences between the two HIT groups, we pooled these groups to increase statistical power, leaving us with 3 groups post intervention; (1) a PCOS Non-Ex group (*n* = 23), (2) a PCOS HIT group (*n* = 41), and (3) a Non-PCOS HIT group (*n* = 15).

We used linear mixed models with participants as random factor and the effect of time and group allocation as fixed effects with these levels: PCOS baseline, Non-PCOS Baseline, PCOS Non-Ex post intervention, PCOS HIT post intervention, and Non-PCOS HIT post intervention. We adjusted for baseline values as recommended by Twisk et al. (2018). Descriptive statistics at baseline are reported as mean ± SD, and comparisons within and between groups are reported as estimated means with 95% confidence intervals. Normality of residuals was evaluated using Q-Q plots. For two of the dependent variables, the residuals were not normally distributed (insulin concentration and HOMA-IR) and logarithmically transformed to obtain normality. As the results were substantially the same after log-transformation, we report the results for the non-transformed variables to improve interpretation. Group means for weekly exercise training sessions for PCOS HIT and Non-PCOS HIT were compared using Student's *t*-test for independent samples, and presented as mean ± SD.

We considered two-sided *P*-values < 0.05 as statistically significant. All analyses were carried out using SPSS version 25.0 (SPSS Inc., United States).

## Results

The distribution of the four PCOS phenotypes (Azziz et al., 2009) were as follows for the Non-Ex and PCOS HIT groups, respectively: 39 and 24% with phenotype A (oligo/amenorrhea + hyperandrogenism + polycystic ovaries), 0 and 15% with phenotype B (oligo/amenorrhea + hyperandrogenism), 39 and 25% with phenotype C (hyperandrogenism + polycystic ovaries), and 22 and 37% with phenotype D (oligo/amenorrhea + polycystic ovaries).

### Comparisons Between Women With and Without PCOS at Baseline

**Figure 2:**
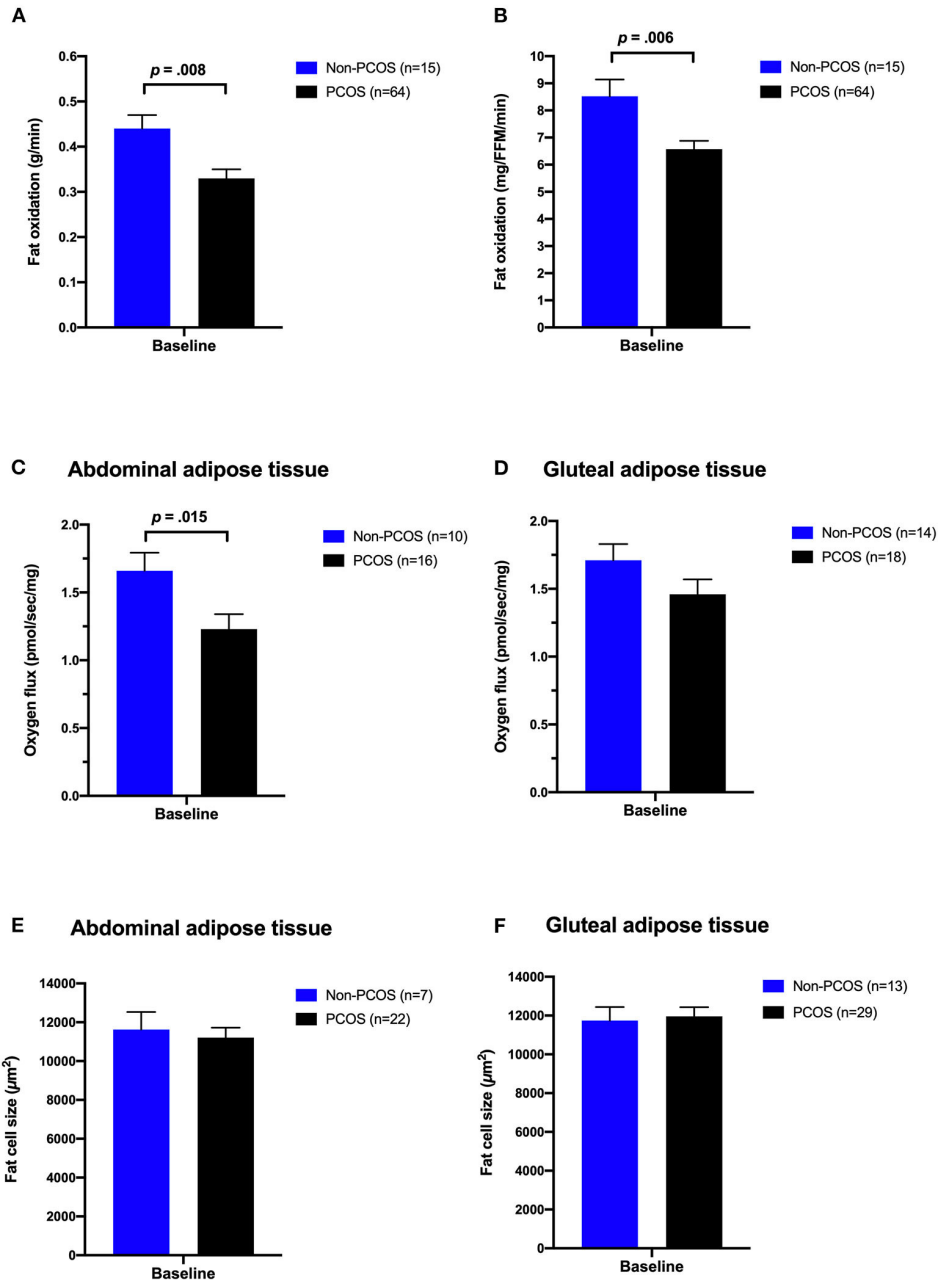
Comparisons between women with PCOS and non-PCOS women at baseline. Whole-body fat oxidation during submaximal exercise **(A,B)**, oxygen flux with complex I + II linked substrates in subcutaneous abdominal **(C)** and gluteal adipose tissue **(D)**, and abdominal **(E)** and gluteal **(F)** adipose tissue cell size in non-PCOS (blue bars) and women with PCOS (black bars). The bars and error bars represent estimated means and SE based on linear mixed models.

Table 1 shows baseline characteristics of the Non-PCOS women and women with PCOS. Women with PCOS had greater waist/hip ratio and lower fat oxidation rates compared with Non-PCOS women (Figures 2A,B). When we compared fat oxidation rates between the 15 women with PCOS who were individually matched with the 15 Non-PCOS women, there were no difference in fat oxidation rates; 7.81 ± 2.14 vs. 8.52 ± 1.26 mg/FFM/min (*p* = 0.28) for women with PCOS and Non-PCOS women, respectively. There was no statistically significant difference in HOMA-IR between the groups, although 43 women with PCOS (68%) vs. five Non-PCOS women (42%) had HOMA-IR ≥ 2.5. Abdominal adipose tissue oxygen flux was higher in Non-PCOS women compared with women with PCOS (Figure 2C). No differences were observed in gluteal adipose tissue oxygen flux (Figure 2D). Nor were there any differences in metabolic outcomes, or abdominal and gluteal adipose tissue cell size between these groups (Figures 2E,F).

**Table 1:** Baseline characteristics of PCOS and non-PCOS women.

	**PCOS**		**Non-PCOS**		***P-values***
	***n***	**Mean ± SD**	***n***	**Mean ± SD**	
Age (years)	64	30 ± 5	15	31 ± 6	0.50
Body weight (kg)	64	85.1 ± 19.6	15	81.2 ± 17.1	0.48
BMI (kg/m^2^)	64	30.5 ± 6.5	15	28.4 ± 5.6	0.24
FFM (kg)	64	50.7 ± 6.8	15	51.0 ± 5.6	0.89
Body fat percentage (%)	64	39.2 ± 8.9	15	34.6 ± 9.7	0.08
Waist circumference (cm)	63	101 ± 17	14	92 ± 13	0.22
Hip circumference (cm)	63	113 ± 14	14	112 ± 12	0.81
Waist/Hip Ratio	63	0.90 ± 0.09	14	0.82 ± 0.05	**0.007**
HbA1c (mmol/mol)	64	32.4 ± 3.2	13	31.3 ± 4.0	0.30
VO_2_peak (mL/min/kg)	64	33.1 ± 7.2	15	36.0 ± 6.8	0.17
VO_2_peak (L/min)	64	2.7 ± 0.4	15	2.9 ± 0.3	0.21
Glucose (mmol/L)	63	5.0 ± 0.5	13	4.8 ± 0.4	0.40
Insulin (pmol/L)	64	117 ± 89	12	84 ± 61	0.28
HOMA-IR	63	4.5 ± 3.8	12	3.0 ± 2.3	0.24
Total testosterone (nmol/L)	64	1.5 ± 0.6		-	
SHBG (nmol/L)	64	43 ± 23		-	

*Values are mean ± SD. PCOS, Polycystic ovary syndrome; BMI, body mass index; FFM, Fat free mass; VO_2_peak, peak oxygen uptake; HOMA-IR, Homeostatic model assessment of insulin resistance; SHBG, Sex Hormone Binding Globulin. Testosterone and SHBG concentrations were only measured in women with PCOS. Statistically significant P-values are in bold*.

### Physiological, Metabolic, and Adipose Tissue Responses to High-Intensity Interval Training

On average, women in the PCOS HIT group completed 2.2 ± 0.5 weekly sessions while women in the Non-PCOS HIT group completed 2.6 ± 0.4 weekly sessions (*p* = 0.025), which corresponds to ~6 exercise sessions less in total over the 16 weeks. Seven women with PCOS and one woman in the Non-PCOS group did not register with Polar Flow, therefore we were unable to include their exercise training data.

Table 2 shows the changes in anthropometric measures, metabolic variables, cardiorespiratory fitness and adipose tissue fat cell size from baseline to after 16 weeks of HIT. The PCOS Non-Ex group reduced body weight and body mass index (BMI) more than PCOS HIT (*p* = 0.021 and *p* = 0.031, respectively), despite no differences in the monitored physical activity level or self-reported daily energy intake (data not shown). Non-PCOS women reduced their body weight and BMI after 16 weeks of HIT, with no between-group differences compared with the PCOS HIT group. Waist circumference and waist/hip ratio decreased in the PCOS HIT group (*p* = 0.011 and *p* = 0.007, respectively), with no between group-differences.

**Table 2:** Effects of 16 weeks of non-exercise or high-intensity interval training.

	**Non-PCOS HIT**		**PCOS Non-Ex**		**PCOS HIT**		**Between PCOS group difference** ** (group x time interaction)**		**Between HIT group difference** ** (group x time interaction)**	
	**Estimate (CI)**	***P***	**Estimate (CI)**	***P***	**Estimate (CI)**	***P***	**Estimate (CI)**	***P***	**Estimate (CI)**	***P***
Body weight (kg)	−1.52 (−2.88 to −0.17)	**0.028**	−2.19 (−3.35 to −1.03)	** <0.001**	−0.44 (−1.36 to 0.49)	0.35	1.75 (0.27 to 3.24)	**0.021**	−1.09 (−2.73 to 0.55)	0.19
BMI (kg/m^2^)	−0.54 (−1.00 to 0.06)	**0.029**	−0.79 (−1.20 to −0.37)	** <0.001**	−0.20 (−0.53 to 0.13)	0.13	0.58 (0.06 to 1.11)	**0.031**	−0.34 (−0.92 to 0.25)	0.25
FFM (kg)	0.32 (−0.36 to 1.00)	0.35	−0.28 (−0.86 to 0.30)	0.33	−0.09 (−0.55 to 0.37)	0.70	0.19 (−0.55 to 0.93)	0.60	0.41 (−0.41 to 1.23)	0.32
Body fat percentage (%)	−0.46 (−1.60 to 0.69)	0.43	−1.03 (−2.01 to −0.05)	**0.04**	−0.29 (−1.07 to 0.49)	0.47	0.74 (−0.51 to 1.99)	0.24	−0.19 (−1.58 to 1.20)	0.79
Waist circumference (cm)	−0.62 (−4.11 to 2.88)	0.73	−0.89 (−3.72 to 1.95)	0.53	−2.96 (−5.19 to −0.72)	**0.011**	−2.07 (−5.65 to 1.52)	0.25	2.35 (−1.81 to 6.51)	0.26
Hip circumference (cm)	−0.46 (−3.05 to 2.14)	0.73	−0.49 (−2.59 to 1.61)	0.64	−0.64 (−2.30 to 1.02)	0.44	−0.15 (−2.82 to 2.51)	0.91	0.15 (−2.93 to 3.24)	0.92
Waist/Hip Ratio	−0.00 (−0.03 to 0.03)	0.92	−0.01 (−0.04 to 0.01)	0.20	−0.03 (−0.04 to −0.01)	**0.007**	−0.01 (−0.04 to 0.02)	0.47	0.02 (−0.01 to 0.06)	0.15
HbA1c (mmol/mol)	0.67 (−0.80 to 2.15)	0.37	0.43 (−0.69 to 1.55)	0.45	1.75 (0.81 to 2.68)	** <0.001**	1.32 (−0.10 to 2.73)	0.07	−1.12 (−2.88 to 0.63)	0.21
VO_2_peak (mL/min/kg)	2.20 (0.76 to 3.65)	**0.003**	0.35 (−0.85 to 1.55)	0.56	1.41 (0.43 to 2.39)	**0.006**	1.06 (−0.48 to 2.59)	0.17	0.81 (−0.94 to 2.56)	0.36
VO_2_peak (L/min)	0.13 (0.02 to 0.23)	**0.016**	−0.06 (−0.15 to 0.02)	0.15	0.11 (0.04 to 0.18)	**0.002**	0.17 (0.06 to 0.28)	**0.002**	0.01 (−0.10 to 0.14)	0.82
Glucose (mmol/L)	0.10 (−0.13 to 0.32)	0.38	−0.09 (−0.26 to 0.08)	0.30	−0.11 (−0.25 to 0.03)	0.11	−0.03 (−0.24 to 0.19)	0.81	0.20 (−0.06 to 0.47)	0.13
Insulin (pmol/L)	−11.5 (−41.3 to 18.3)	0.44	−25.1 (−47.2 to −3.0)	**0.026**	−13.6 (−31.6 to 4.4)	0.14	11.5 (−16.5 to 39.4)	0.42	4.1 (−30.8 to 39.1)	0.81
HOMA-IR	−0.35 (−1.72 to 1.01)	0.61	−1.10 (−2.11 to −0.10)	**0.032**	−0.66 (−1.48 to 0.16)	0.11	0.44 (−0.83 to 1.71)	0.49	0.39 (−1.21 to 1.99)	0.63

*Estimated mean difference from baseline values(95% confidence interval) based on linear mixed models*.

*PCOS, Polycystic ovary syndrome; CI, Confidence interval; BMI, body mass index; FFM, Fat free mass; VO_2_peak, peak oxygen uptake; HOMA-IR, Homeostatic model assessment of insulin resistance. Statistically significant P-values are in bold*.

**Figure 3:**
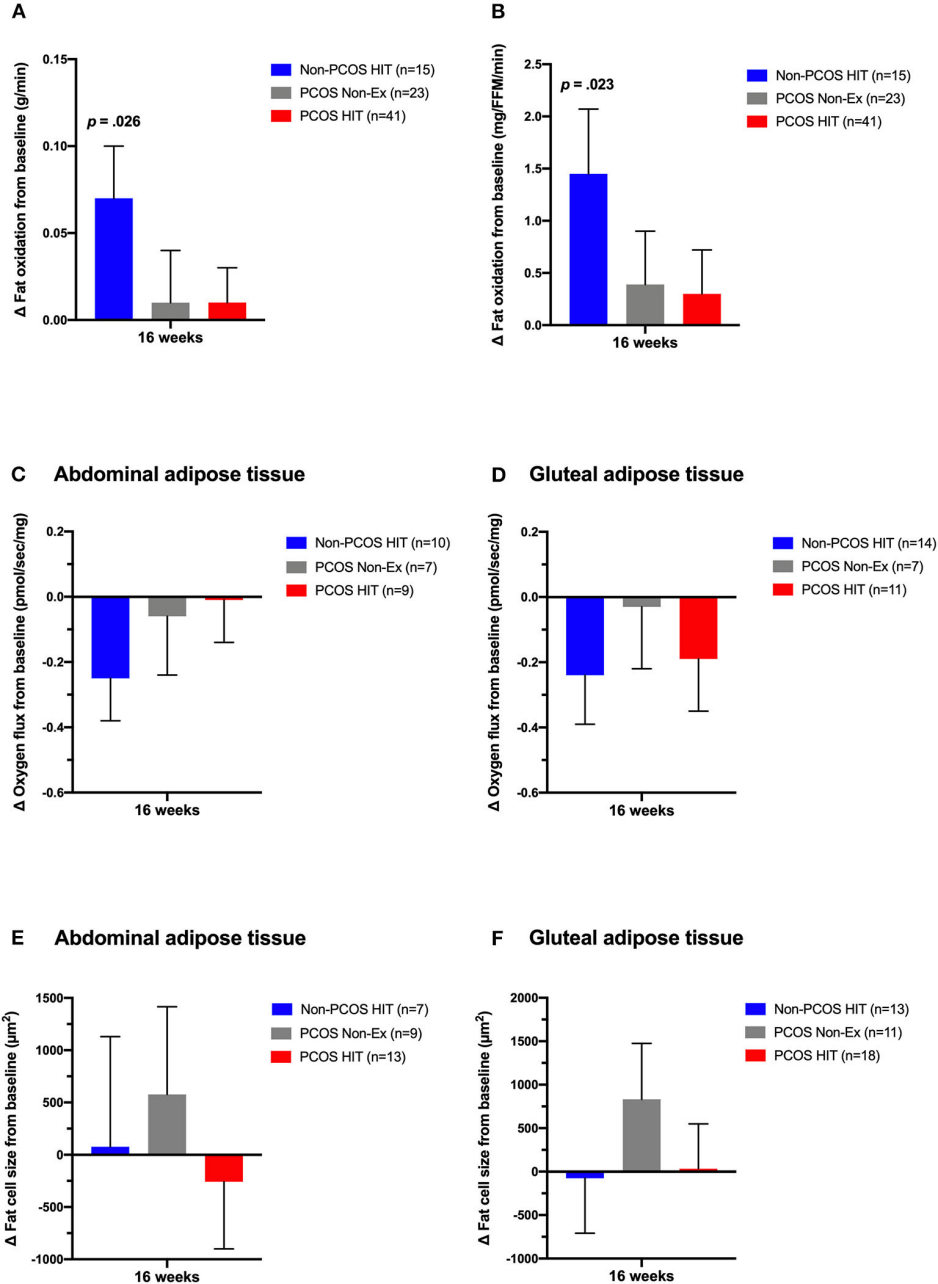
Effects of 16 weeks of non-exercise or high-intensity interval training. Whole-body fat oxidation during submaximal exercise **(A,B)**, oxygen flux with complex I + II linked substrates in subcutaneous abdominal **(C)** and gluteal adipose tissue **(D)**, and abdominal **(E)** and gluteal **(F)** adipose tissue cell size in Non-PCOS HIT (blue bars), PCOS Non-Ex (gray bars), and PCOS HIT (red bars) after the 16 weeks intervention. The bars and error bars represent estimated means and SE based on linear mixed models. *P-*values are for within-group comparisons after 16 weeks of high-intensity interval training.

Cardiorespiratory fitness increased by ~6% in the Non-PCOS HIT group (*p* = 0.003) and ~4% in the PCOS HIT group (*p* = 0.006) after 16 weeks of HIT. The PCOS HIT group improved their absolute VO_2_peak (L/min), but not relative (mL/min/kg), significantly more than the PCOS Non-Ex group (*p* = 0.002). Fat oxidation rates increased in the Non-PCOS HIT group, with no change in the PCOS HIT or PCOS Non-Ex group (Figures 3A,B). There were no between-group differences in changes in oxidation rates after 16 weeks.

We observed no between-group differences in metabolic outcomes, abdominal and gluteal adipose tissue oxygen flux (Figures 3C,D), or cell size (Figures 3E,F) after 16 weeks of HIT.

## Discussion

Our findings provide new data on whole-body fat oxidation during submaximal exercise, and gluteal and abdominal adipose tissue mitochondrial respiration and cell size in women with PCOS and Non-PCOS women, along with the effects of 16 weeks of HIT on these outcomes. We report that 16 weeks of HIT improved fat oxidation rates during submaximal exercise in Non-PCOS women, but not in women with PCOS, despite similar improvements in VO_2_peak. Furthermore, women with PCOS had lower mitochondrial respiration with substrates for complex I + II in subcutaneous abdominal adipose tissue compared to Non-PCOS women. Mitochondrial respiration in both adipose tissue sites were unaffected by 16 weeks of HIT in women with and without PCOS. Finally, subcutaneous gluteal or abdominal adipose tissue cell size did not differ between women with PCOS and Non-PCOS women and 16 weeks of HIT did not alter adipocyte sizes in any group of women.

We report novel findings on absent exercise-induced improvements in fat oxidation during submaximal exercise in women with PCOS, despite improved VO_2_peak, suggesting metabolic inflexibility in women with PCOS. Conversely, fat oxidation rates improved in Non-PCOS women after 16 weeks of HIT. A recent systematic review reported metabolic inflexibility in the rested, insulin-stimulated state in women with PCOS compared with healthy women (Rimmer et al., 2020). Metabolic inflexibility has been linked to insulin resistance and type 2 diabetes (Goodpaster and Sparks, 2017), and Broskey and colleagues found that women with PCOS and obesity (28.8 ± 4.7 years) were as metabolically inflexible as middle-aged women with type 2 diabetes and obesity (58.2 ± 9.9 years) (Broskey et al., 2018). Metabolic inflexibility has also been reported in lean women with PCOS (Hansen et al., 2019) and adolescent girls with PCOS (Kim et al., 2018), suggesting that metabolic inflexibility is associated with PCOS independent of BMI and age.

Similar to our findings in Non-PCOS women, previous studies reported improvements in whole-body fat oxidation during submaximal exercise at 60% of VO_2_peak in healthy recreationally active women after seven sessions of HIT (10 x 4 min bouts at 90% of VO_2_peak separated by 2 min of rest) (Talanian et al., 2007), and in untrained recreationally active men and women after 6 weeks of HIT (using the same HIT protocol as Talanian et al., 2007 but for 3 days/week for 6 weeks) (Perry et al., 2008). In our study, the Non-PCOS women exercised significantly more on a weekly basis compared to the women with PCOS (2.6 ± 0.4 vs. 2.2 ± 0.5 weekly HIT sessions, corresponding to ~6 HIT sessions more in total over the 16 weeks), which may explain the difference observed in training-induced improvements in fat oxidation rates. However, we report similar improvements in VO_2_peak in women with PCOS and Non-PCOS women after 16 weeks of HIT, which suggests that the six fewer HIT sessions performed by women with PCOS cannot fully explain the difference in training-induced improvements in fat oxidation rates. Absent training-induced improvements in women with PCOS have been reported previously; Hansen and colleagues observed improved insulin sensitivity measured by the gold-standard hyperinsulinaemic-euglycemic clamp in healthy women without PCOS, but not in women with PCOS after 14 weeks of exercise training (three weekly exercise sessions; two aerobic HIT sessions and one strength training session) (Hansen et al., 2020). Similarly, Hansen and colleagues reported improved incremental area under the oral glucose tolerance test curve for plasma glucose and insulin after exercise training in healthy women, but not in women with PCOS. Their data suggested that the lack of improvements in insulin action after exercise training were due to impaired ability to upregulate glucose uptake in skeletal muscle.

Skeletal muscle and adipose tissue are crucial tissues in energy metabolism and play a major role in metabolic flexibility in humans. However, little research has been undertaken on metabolic flexibility of white adipose tissue. Metabolic flexibility is driven by cellular processes that may be linked to the mitochondria (Goodpaster and Sparks, 2017). We are the first to explore subcutaneous abdominal and gluteal adipose tissue mitochondrial respiration in women with and without PCOS and the responses to HIT. In our study, we observed higher mitochondrial respiration through complex I + II in subcutaneous abdominal adipose tissue from Non-PCOS women compared to women with PCOS at baseline, no such differences were seen in gluteal adipose tissue. Low-grade inflammation may be one mechanism causing the lower mitochondrial respiration in women with PCOS compared to Non-PCOS women at baseline. There is a proposed link between inflammation and mitochondrial dysfunction in adipocytes, in which the proinflammatory response of macrophages may promote mitochondrial dysfunction in adipocytes (Woo et al., 2019). No changes were observed in mitochondrial respiration in either of the adipose tissue depots in either group of women after 16 weeks of HIT, which suggests that mitochondrial respiration through complex I + II in adipose tissue cannot explain the improved fat oxidation rates and metabolic flexibility observed in Non-PCOS women. Our findings are supported by data from Larsen and colleagues, who showed increased VO_2_peak but no change in mitochondrial respiration in subcutaneous abdominal adipose tissue in overweight but otherwise healthy men and women after 6 weeks of LV-HIT (3 days/week with 5 × 60 s maximal effort work-bouts) (Larsen et al., 2015). However, there are some indications in the literature for exercise-induced changes in adipose tissue mitochondrial respiration. Dohlmann et al. (2018) found decreased mitochondrial respiration in subcutaneous abdominal adipose tissue in healthy men and women after 6 weeks of LV-HIT (18 HIT sessions with 7 × 1 min exercise bouts at ~100% of VO_2_peak), while Mendham et al. (2020) reported increased mitochondrial respiration in subcutaneous abdominal adipose tissue in black South-African women with obesity after 12 weeks of combined aerobic and resistance exercise training (4 days/week of 40–60 min with aerobic exercise at 75–80% HR_peak_ and resistance training that included upper- and lower-body exercises at 60–70% HR_peak_). We are not sure of the reasons for these divergent findings, but they may be explained by different exercise modalities and intensities.

We did not observe changes in mitochondrial respiration in adipose tissue after 16 weeks of HIT. We speculate that changes may have occurred in skeletal muscle mitochondrial function as Larsen and colleagues have previously reported increased mitochondrial respiration in skeletal muscle, but not in abdominal adipose tissue, after 6 weeks of HIT in men and women who were overweight but otherwise healthy (Larsen et al., 2015). Possible gains, or lack of gains, in skeletal muscle mitochondrial function could explain the improved whole-body fat oxidation during submaximal exercise in Non-PCOS women, and lack of improvement in women with PCOS after 16 weeks of HIT. However, we did not obtain any muscle biopsies in our study.

We observed no differences in subcutaneous abdominal or gluteal fat cell size between Non-PCOS women and women with PCOS, nor do we report changes in cell size after 16 weeks of HIT in either group of women. A case-control study on women with PCOS and age- and BMI-matched women without PCOS reported enlarged abdominal adipocyte volumes in women with PCOS, and an association between hypertrophic adipocytes and insulin resistance (Manneras-Holm et al., 2011). The divergent findings in our study and the study by Manneras-Holm may be explained by higher power in their study (27 women in both groups).

Similar to our findings, previous studies have reported no changes in subcutaneous abdominal adipose tissue volume and size after 12 weeks of exercise training in men with overweight or obesity (3 days/week; aerobic exercise twice/week cycling for 30 min at 70% maximal watt and resistance training once weekly) (Stinkens et al., 2018), or after 16 weeks of exercise in women with PCOS (3 days/week for 30 min at an intensity of faster than normal walking pace) (Stener-Victorin et al., 2012). A longer and more intense training program may be required to change adipose tissue structure. Després and colleagues observed a sex difference in the sensitivity to exercise training, with less responsiveness in female adipose tissue. Twenty weeks of endurance training for 40 min at 80% HR_max_ 4–5 times weekly did not influence fat percentage or adipose cell weight in women, whereas males showed reductions in both fat percentage and adipose cell weight (Despres et al., 1984).

Our data indicate that women with PCOS allocated the non-exercising control group most likely introduced some lifestyle changes during the study period as we observed reductions in body mass, BMI and body fat percentage and improvements in fasting insulin concentration and HOMA-IR in this group. Although we detected no changes in self-reported daily energy intake or physical activity level, these were only recorded for a brief period of time (4–5 days at each timepoint). At study inclusion, women with PCOS assigned to the control group were informed about the current recommendations of at least 150 min weekly of moderate intensity physical activity, which could have affected their physical activity behavior. Furthermore, individuals who volunteer for exercise studies usually hope to be allocated to the exercise intervention, and women allocated to the control group have most likely been motivated to introduce lifestyle changes.

Subgroup analyses from a systematic review and meta-analysis on the effectiveness of exercise compared to control in women with PCOS showed greater improvements in cardiometabolic outcomes in supervised vs. unsupervised exercise interventions (Kite et al., 2019), and a supervised rather than semi-supervised exercise protocol in our study could possibly have resulted in greater gains for women with (and without) PCOS. Furthermore, including a more varied exercise protocol with a combination of LV-HIT and HV-HIT sessions could have induced larger improvements, as shown previously (Almenning et al., 2015).

Major strengths of our study are the pair-wise age- and BMI-matching of women with PCOS and Non-PCOS women, the rigorous inclusion and exclusion criteria, and that we explored two adipose tissue depots in women with and without PCOS. We also acknowledge study limitations. We used different methodologies to estimate body composition at the two study centers, limiting the baseline comparison for these outcomes between women with and without PCOS. The Inbody 720 scale that we used for participants in Norway underestimates body fat and overestimates FFM compared with DXA (Mclester et al., 2018). Due to limited adipose tissue biopsy volumes, we were unable to compare mitochondrial respiration and fat cell size between the women with PCOS and Non-PCOS women who were individually age- and BMI-matched. Furthermore, due to limited adipose tissue volumes and statistical power, we were unable to run meaningful statistical analyses for LV-HIT and HV-HIT groups, which made us unable to detect differences in the effects of LV-HIT and HV-HIT on mitochondrial respiration and fat cell size. Instead, data for LV-HIT and HV-HIT were pooled to increase the statistical power and to investigate the effect of HIT.

In conclusion, we observed exercise-induced improvements in whole-body fat oxidation during submaximal exercise in Non-PCOS women but not in women with PCOS after 16 weeks of HIT. These findings suggest metabolic inflexibility in women with PCOS. Mitochondrial respiration was lower in subcutaneous abdominal, but not gluteal, adipose tissue in women with PCOS compared to Non-PCOS women, and 16 weeks of HIT did not alter adipose tissue mitochondrial respiration in either group of women. Further studies are required to ascertain the underlying mechanisms behind the absent exercise-induced improvements in fat oxidation and metabolic inflexibility observed in women with PCOS.

## Data Availability Statement

The raw data supporting the conclusions of this article will be made available by the authors, without undue reservation.

## Ethics Statement

The studies involving human participants were reviewed and approved by The Regional Committee for Medical and Health Research Ethics in Central Norway and The ACU Human Research Ethics Committee. The patients/participants provided their written informed consent to participate in this study.

## Author Contributions

SLi drafted the manuscript. SLi, RR, and SLa analyzed the data. SLi and SLy performed statistical analyses. SLi, IK, and TM were responsible for study conception and design, coordinated the studies at the two sites, performed measurements on testing days, and supervised the exercise training. All authors provided feedback and approved the final manuscript.

## Conflict of Interest

The authors declare that the research was conducted in the absence of any commercial or financial relationships that could be construed as a potential conflict of interest.
